# Typical curve with G1 constraints for curve completion

**DOI:** 10.1186/s42492-021-00095-9

**Published:** 2021-11-26

**Authors:** Chuan He, Gang Zhao, Aizeng Wang, Fei Hou, Zhanchuan Cai, Shaolin Li

**Affiliations:** 1 School of Mechanical Engineering and Automation, Beihang University, Beijing, 100191 China; 2 State Key Laboratory of Lunar and Planetary Sciences, Macau University of Science and Technology, Macau, 999078 China; 3 State Key Laboratory of Virtual Reality Technology and Systems, Beihang University, Beijing, 100191 China; 4 State Key Laboratory of Computer Science, Institute of Software, Chinese Academy of Sciences, Beijing, 100190 China; 5 State Key Laboratory of Computer Science, Institute of Software, University of Chinese Academy of Sciences, Beijing, 100049 China; 6 Faculty of Information Technology, Macau University of Science and Technology, Macau, 999078 China

**Keywords:** Typical curves, Monotonic curvature, G1 interpolation, Curve completion, Euler spiral

## Abstract

This paper presents a novel algorithm for planar G1 interpolation using typical curves with monotonic curvature. The G1 interpolation problem is converted into a system of nonlinear equations and sufficient conditions are provided to check whether there is a solution. The proposed algorithm was applied to a curve completion task. The main advantages of the proposed method are its simple construction, compatibility with NURBS, and monotonic curvature.

## Introduction

**Fig. 1:**
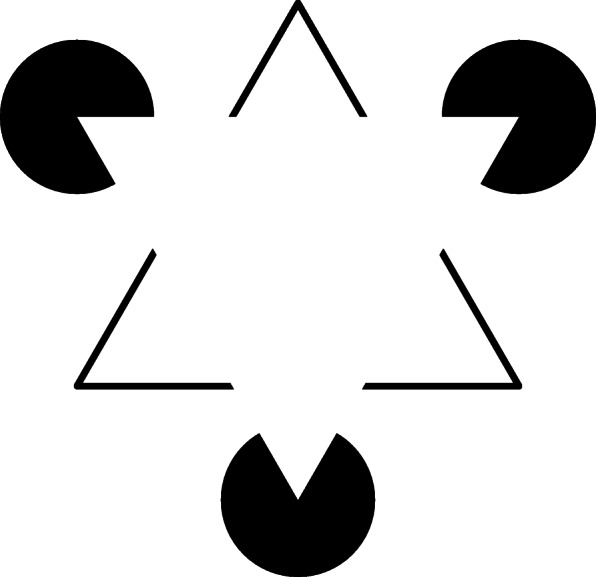
Kanizsa triangle consisting of three discs, each of which is missing a triangular section, and three pairs of lines. Most humans will have the impression that there is an upright triangle with black edges and an inverted triangle with white edges, which do not really exist

G1 interpolation is an essential problem in many applications such as path planning and curve completion. The goal is to find a transition curve that matches the positions and associated unit tangent vectors at two given endpoints [1]. Curve completion aims to find a pleasing contour to fill in an object boundary that is partially occluded [2, 3]. This process differs significantly for humans and computers. For example, the visual system of humans can automatically complete missing parts of curves (Fig. 1), which is called visual curve completion [4–6]. For computers, it is necessary to identify a fairing curve matching “boundary conditions” among potential transition curves, which is called curve completion.

In computer-aided design (CAD) and computer-aided geometric design, the measurement of the fairness of curves typically depends on the curvature distribution. Farin and Sapidis [7] assumed that a fairing curve should have relatively few monotonic curvature variation segments. By virtue of linear curvature variation and the ability to minimize changes in the total curvature, the Euler spiral is considered to be the most pleasing curve for shape completion [2], so it has been widely used in various applications such as path planning and curve completion [2, 8–11]. However, the Euler spiral is defined in the form of a transcendental function that is computationally intensive and inefficient. Furthermore, the Euler spiral is not compatible with the NURBS methods, which are the standard methods in existing CAD software [12].

To overcome these shortcomings, some methods using polynomial curves or arc splines to approximate Euler curves have been proposed [3, 13–16]. Another solution is to use a polynomial curve as a design object directly. In highway design, a transition curve is typically defined between a line and a circular curve or between two circular curves. Walton et al. [17–19] used planar cubic Bézier spirals to construct transition curves with G2 continuity for different boundary constraints and the results of refs. [17, 19] were extended in refs. [20, 21]. Traditional methods for designing Bézier curves with monotonic curvature focused on adjusting the positions of control points until Mineur proposed the concept of a typical curve [22], which indicates a special subset of planar Bézier curves with control edges that maintain a specific geometric constraint. Sánchez-Reyes [23] pointed out that these constrained typical curves belong to the family of offset rational sinusoidal spirals and can also be expressed as a subset of rational Bézier curves called p-Bézier curves [24]. Recently, Wang et al. [25] proposed a geometric proof of the necessary and sufficient conditions for the curvature monotonicity of typical curves. In 2006, Farin [26] extended typical curves to 3D class-A Bézier curves, whose control edges were generated from a class-A matrix and initial control edge. However, the conditions of the class-A matrix have been proven to be incomplete [27] and several counterexamples have been reported [28]. Yoshida et al. [29] proposed an interactive method to generate general class-A Bézier curves by perturbing the elements of a planar typical class-A matrix. Furthermore, typical curves converge to logarithmic spiral segments as the degree is elevated and the drawable regions of typical curves for boundary constraints under different degrees can be obtained.

In recent years, geometric continuity has also been discussed based on trigonometric Bézier curves with shape parameters [30–33]. Generalized trigonometric Bézier curves are flexible and can achieve G2 continuity. However, these methods are more complex than polynomials and have not provided a way to obtain a monotonic variation of curvature. This study analyzed the sufficient conditions for the G1 interpolation problem using typical curves and developed a novel algorithm to find a fairing solution, which is applied to curve completion. For a given set of constraints, multiple solutions may exist, so a suitable criterion must be defined to find the optimal solution automatically. Furthermore, a designer can modify data manually to obtain a fair solution to fit different situations. The main contributions of this work can be summarized as follows. The sufficient conditions for G1 interpolation based on typical curves are provided and curve completion using Bézier curves with monotonic curvature is realized.

The remainder of this paper is organized as follows. Typical curves Section introduces typical curves comprehensively. Methods Section discusses the G1 interpolation problem, provides sufficient conditions for a typical curve solution, and presents a novel algorithm to solve G1 boundary constraints. In Results and discussion Section, some examples are presented to demonstrate the practicability and superiority of the proposed algorithm. The method is then applied in the application of curve completion. Finally, this paper is concluded in Conclusions Section.

## Typical curves

A planar Bézier curve of degree *k* can be expressed as

1 $$ \boldsymbol{P}(t)=\sum \limits_{i=0}^k{\boldsymbol{b}}_i{B}_{i,k}(t),t\in \left[0,1\right] $$

where ***b***_*i*_ are the two-dimensional control points and *B*_*i*, *k *_(*t*) is the *i*-th Bernstein polynomial of degree *k*. Let the forward difference vector ***V***_*i*_ = ***b***_*i* + 1_ − ***b***_*i *_(*i* = 0, …, *k* − 1) be the control edge. Then, a typical curve is obtained with control edges satisfying

2 $$ {\boldsymbol{V}}_i=s\cdot {\boldsymbol{R}}_{\theta}\cdot {\boldsymbol{V}}_{i-1} $$

where *s* is a positive scale factor and ***R***_*θ*_ is a second-order rotation matrix with a rotation angle $\theta \in \left[-\frac{\uppi}{2},\frac{\uppi}{2}\right]$. A *k*-degree typical curve exhibits monotonic curvature variation if and only if

3 $$ \left\{\begin{array}{l}s\cdot \cos \theta \ge 1,s\ge 1\\ {}s\le \cos \theta, 0<s<1\ \end{array}\right. $$

**Fig. 2:**
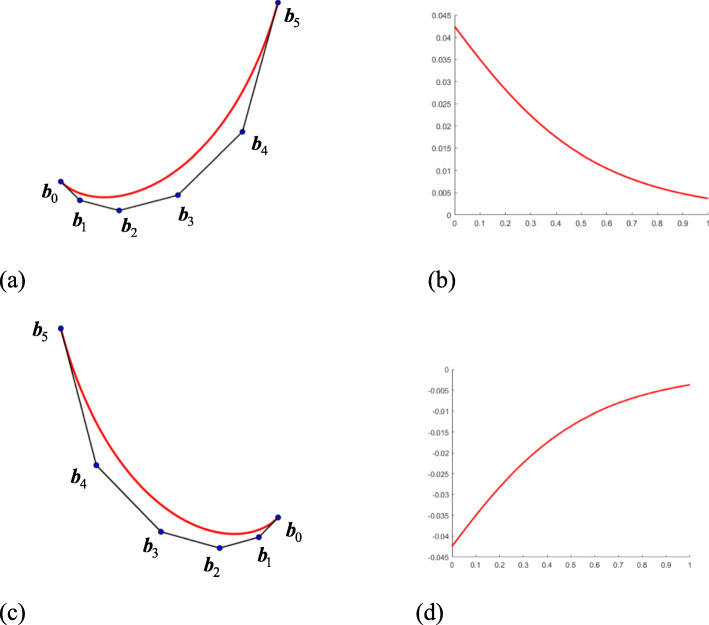
The differences between different rotation angles with opposite signs. **a**: $\theta \in \left[0,\frac{\uppi}{2}\right]$, *s* > 1, and *s* ⋅ cos *θ* ≥ 1; **b**: The curvature is positive and decreases; **c**: $\theta \in \left[-\frac{\uppi}{2},0\right]$, *s* > 1, and *s* ⋅ cos *θ* ≥ 1; (d): The curvature is negative and increases

When $\theta \in \left[0,\frac{\uppi}{2}\right]$, it is assumed that the control edge rotates counterclockwise (Fig. 2(a)) and the relative curvature is assumed to be positive (Fig. 2(b)). In this case, if the scale factor *s* > 1, then the curve ***P***(*t*) has a monotonically decreasing curvature. If 0 < *s* < 1, then ***P***(*t*) has a monotonically increasing curvature. When $\theta \in \left[-\frac{\uppi}{2},0\right]$, it is assumed that the control edge rotates clockwise (Fig. 2(c)) and the relative curvature is negative (Fig. 2(d)), so the monotonicity of curvature is opposite to $\theta \in \left[0,\frac{\uppi}{2}\right]$. As a result of the construction process of control edge vectors, the generated curve can only be ‘C’ shaped rather than ‘S’ shaped.

## Methods

This section presents the G1 interpolation algorithm based on typical curves. First, the G1 interpolation problem based on typical curves is introduced briefly. The given endpoint-orientation pairs are then divided into two cases, and a novel algorithm for constructing a typical curve under the given boundary constraints is proposed.

### G1 Hermite interpolation problem

In many interpolation problems, not only positions, but also the corresponding derivative values or higher-order derivatives at endpoints are required to be equal, which is called Hermite interpolation. If the interpolation curve matches the given unit tangent vectors at the two endpoints, it is a two-point G1 Hermite interpolation. Additionally, if the given curvatures at the two endpoints are also matched, it is called two-point G2 Hermite interpolation [1].

Although G2 interpolation has better continuity, it also introduces stronger constraints for the interpolation curve and the high-order continuity at endpoints is sometimes difficult to guarantee. In some applications, G1 interpolation is preferable to G2 interpolation when considering cost effectiveness, such as curve completion or path planning using Euler spirals [1]. Consider two different points ***P***_*A*_ = (*x*_*A*_, *y*_*A*_)^T^ and ***P***_*B*_ = (*x*_*B*_, *y*_*B*_)^T^ with the corresponding unit tangent vectors ***T***_*A*_ = (cos*θ*_*A*_, sin*θ*_*A*_)^T^ and ***T***_*B*_ = (cos*θ*_*B*_, sin*θ*_*B*_)^T^, where the three vectors ***T***_*A*_, ***T***_*B*_, and ***P***_*A*_***P***_*B*_ = ***P***_*B*_ − ***P***_*A*_ are not all parallel. G1 Hermite interpolation is used to find a fairing curve that joins ***P***_*A*_ and ***P***_*B*_ on the condition that the tangent vectors at the endpoints match ***T***_*A*_ and ***T***_*B*_, respectively.

Walton et al. [1, 34] proposed an improved Euler spiral algorithm for shape completion. However, the Euler spiral is not compatible with NURBS. Therefore, the paper proposes a more intuitive algorithm for the G1 interpolation problem based on a typical curve, which is easier to construct.

### G1 interpolation problem based on a typical curve

#### Proposition 1

Consider the positions of two endpoints ***P***_*A*_ = (*x*_*A*_, *y*_*A*_)^T^ and ***P***_*B*_ = (*x*_*B*_, *y*_*B*_)^T^, and their associated unit tangent vectors ***T***_*A*_ = (cos*θ*_*A*_, sin*θ*_*A*_)^T^ and ***T***_*B*_ = (cos*θ*_*B*_, sin*θ*_*B*_)^T^, where *θ*_*A*_ and *θ*_*B*_ are orientation angles with multiple values. When *θ*_*A*_ or *θ*_*B*_ are increased or reduced by 2*m* ⋅ π(*m* ∈ Z), the unit tangent vector remains unchanged. The sufficient condition for the existence of a *k*-degree typical curve matching the boundary constraints is that the following equation has real number solutions satisfying *s* ⋅ cos *θ* ≥ 1 (*s* ≥ 1) or *s* ≤ cos *θ* (0 < *s* < 1):

4 $$ \left\{\begin{array}{l}{x}_A+\sum \limits_{i=1}^k\left\Vert {\boldsymbol{V}}_0\right\Vert \cdot {s}^{i-1}\cdot \cos \left[{\theta}_A+\left(i-1\right)\cdot \theta \right]={x}_B\\ {}{y}_A+\sum \limits_{i=1}^k\left\Vert {\boldsymbol{V}}_0\right\Vert \cdot {s}^{i-1}\cdot \sin \left[{\theta}_A+\left(i-1\right)\cdot \theta \right]={y}_B\end{array}\right. $$

where ‖***V***_0_‖ and *s* are free variables with positive values and *θ* = (*θ*_*B*_ − *θ*_*A*_)/(*k* − 1).

#### Proof

When the degree *k* is fixed, *θ* = (*θ*_*B*_ − *θ*_*A*_)/(*k* − 1) is the rotation angle of the typical curve. If there are real number solutions such that *s* ⋅ cos *θ* ≥ 1 (*s* ≥ 1) or *s* ≤ cos *θ* (0 < *s* < 1), which are the sufficient and necessary conditions for the monotonic curvature of a typical curve, the curvature of the Bézier curve constructed using this method must be monotonous.

Now we are going to analyze the sufficient conditions for the existence of solutions for Eq. (4). Considering the degree *k* as a free variable in Eq. (4), there are three free variables *k* ∈ *Z*^+^, *s* > 0, and ‖***V***_0_‖ > 0. Eliminating ‖***V***_0_‖ yields

5 $$ \sum \limits_{i=1}^k{s}^{i-1}\cdot \left[\left({y}_B-{y}_A\right)\cdot \cos \left({\theta}_A+\left(i-1\right)\cdot \theta \right)-\left({x}_B-{x}_A\right)\cdot \sin \left({\theta}_A+\left(i-1\right)\cdot \theta \right)\right]=0 $$

**Fig. 3:**
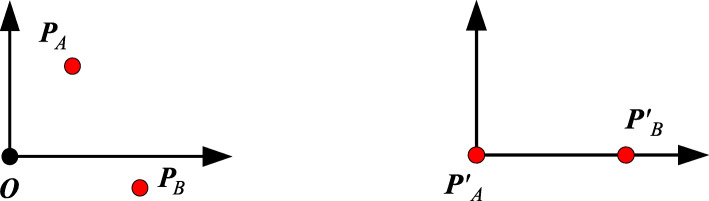
Pre-transformation of arbitrary given endpoints such that ***P***'_*A*_ = (0, 0)^T^ and ***P***'_*A*_***P***'_*B*_ denotes the positive direction of the *x* axis. This is essentially just a transformation of the local coordinate system. **a**: Arbitrary given endpoints; **b**: After pre-transformation

Because Bézier curves are invariant under affine transformations, one can place the first endpoint ***P***_*A*_ = (*x*_*A*_, *y*_*A*_)^T^ at the origin and the second endpoint ***P***_*B*_ = (*x*_*B*_, *y*_*B*_)^T^ at the positive half of the *x* axis using a pre-transformation, which leads to *x*'_*A*_ = 0, *y*'_*A*_ = 0, *x*'_*B*_ > 0 and *y*'_*B*_ = 0 (Fig. 3). Then, Eq. (5) is converted into

6 $$ \sum \limits_{i=1}^k{s}^{i-1}\cdot \left[-x{\hbox{'}}_B\cdot \sin \left(\theta {\hbox{'}}_A+\left(i-1\right)\cdot \theta \right)\right]=0 $$

The superscript ' indicates a new variable obtained from the pre-transformation. The pre-transformation can be used to construct a new local coordinate system. Equation (6) is a polynomial of degree *k* and one can infer the number of positive roots from the Descartes rule of signs [35]. The number of sign changes in the coefficients is equal to the number of sign changes in sin[*θ*'_*A*_ + (*i* − 1) ⋅ *θ*], *i* = 1, …, *k*. Therefore, a sufficient condition to guarantee that Eq. (6) has at least one positive solution is that sin[*θ*'_*A*_ + (*i* − 1) ⋅ *θ*] changes signs an odd number of times, meaning that after pre-transformation, the sign of the sine angle for all control edges changes an odd number of times.

#### Angle sign change condition

Consider ***P***_*A*_ as the origin and ***P***_*A*_***P***_*B*_ as the positive direction of the *x* axis. In this new local coordinate system, the function sin*α* (*θ*'_*A*_ ≤ *α* ≤ *θ*'_*B*_) only changes its sign an odd number of times.

#### Proposition 2

If the ASC condition is satisfied, then one can always find an appropriate degree *k* such that the solutions of Eq. (4) or (6) satisfy Eq. (3).

#### Proof

Because the affine transformation does not change the solution of the curve, Eqs. (4, 6) have the same solution and the ASC condition guarantees a positive solution for Eq. (4). When the degree *k* gradually increases in Eq. (6) and the rotation angle $\theta =\frac{\theta_B-{\theta}_A}{k-1}\to 0$ cos*θ* → 1, there is an *s*_*k*_ > 0 such that *s*_*k*_ ⋅ cos *θ* ≥ 1 (*s*_*k*_ ≥ 1) or *s*_*k*_ ≤ cos *θ* (0 < *s*_*k*_ < 1), meaning the solutions of Eq. (4) or (6) satisfy Eq. (3).

#### Corollary 1

For Eq. (4) or (6), if there is a typical curve satisfying the constraints when *k* = *m* ≥ 2, then there are also typical curve solutions when *k* ≥ *m* + 1. This means that the G1 interpolation problem has multiple solutions consisting of typical curves and the limit of the solutions increases *θ* → 0 with an increase in the degree *k*.

The ASC condition is a sufficient condition for the positive solution of Eq. (6). Even if the ASC condition is not satisfied, Eq. (4) or (6) may still have a positive root. In contrast, the ASC condition cannot guarantee solutions satisfying Eq. (3) under a fixed degree.

### Two cases of G1 constraints

For convenience of expression, homogeneous coordinates will be used to represent points and vectors for the remainder of this paper. For a 2D point (*x*, *y*)^T^, its homogeneous coordinate can be written as (*ωx*, *ωy*, *ω*)^T^, where *ω* ≠ 0. This study used *ω* = 1. For a 2D vector (*x*, *y*)^T^, the homogeneous coordinate can only be expressed as (*x*, *y*, 0)^T^.

For an arbitrary ***P***_*A*_ = (*x*_*A*_, *y*_*A*_, 1)^T^, ***P***_*B*_ = (*x*_*B*_, *y*_*B*_, 1)^T^, ***T***_*A*_ = (cos*θ*_*A*_, sin*θ*_*A*_, 0)^T^, and ***T***_*B*_ = (cos*θ*_*B*_, sin*θ*_*B*_, 0)^T^, one can divide the G1 constraints into two cases according to the relationship between the relative positions of the three vectors ***T***_*A*_ = (cos*θ*_*A*_, sin*θ*_*A*_, 0)^T^, ***T***_*B*_ = (cos*θ*_*B*_, sin*θ*_*B*_, 0)^T^, and ***P***_*A*_***P***_*B*_ = (*x*_*B*_ − *x*_*A*_, *y*_*B*_ − *y*_*A*_, 0)^T^. This analysis does not consider the degenerate case in which all three vectors are parallel.

**Fig. 4:**
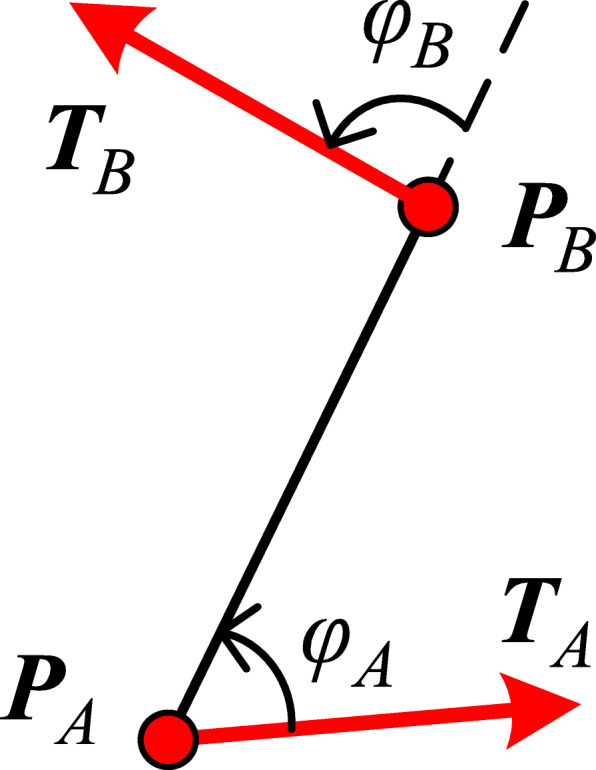
Angle from vector ***T***_*A*_ to vector ***P***_*A*_***P***_*B*_, and from ***P***_*A*_***P***_*B*_ to ***T***_*B*_

Let −π ≤ *φ*_*A*_ ≤ π denote the angle from vector ***T***_*A*_ to vector ***P***_*A*_***P***_*B*_ (Fig. 4). The sign of *φ*_*A*_ indicates the direction and *φ*_*A*_ > 0 indicates that ***T***_*A*_ is on the right side of ***P***_*A*_***P***_*B*_. Similarly, −π ≤ *φ*_*B*_ ≤ π denotes the angle from vector ***P***_*A*_***P***_*B*_ to vector ***T***_*B*_.

**Fig. 5:**
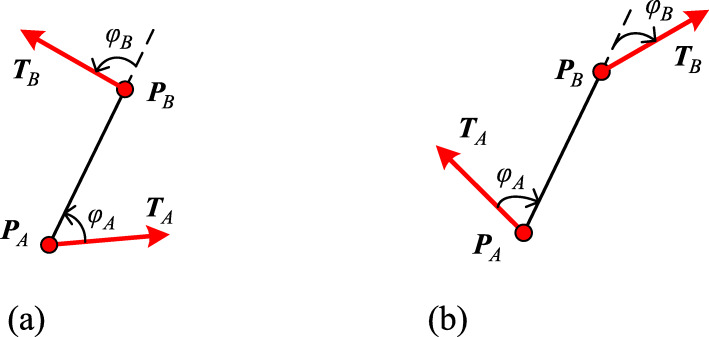
Case I: *φ*_*A*_ ⋅ *φ*_*B*_ > 0. **a**: *φ*_*A*_ > 0, *φ*_*B*_ > 0; **b**: *φ*_*A*_ < 0, *φ*_*B*_ < 0

Considering the signs of these two angles, one can divide all potential constraints into two cases as follows:

**Case I** *φ*_*A*_ ⋅ *φ*_*B*_ > 0, meaning ***T***_*A*_ and ***T***_*B*_ are located on opposite sides of ***P***_*A*_***P***_*B*_ (Fig. 5).

**Fig. 6:**
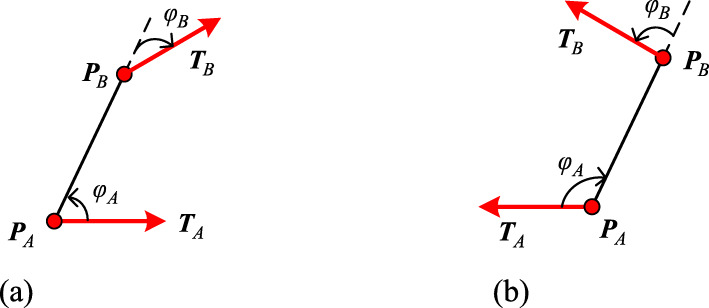
Case II: *φ*_*A*_ ⋅ *φ*_*B*_ < 0. **a**: *φ*_*A*_ > 0, *φ*_*B*_ < 0; **b**: *φ*_*A*_ < 0, *φ*_*B*_ > 0

**Case II** *φ*_*A*_ ⋅ *φ*_*B*_ < 0, meaning that ***T***_*A*_ and ***T***_*B*_ are located on the same side of ***P***_*A*_***P***_*B*_ (Fig. 6).

Because *θ*_*A*_ and *θ*_*B*_ can be increased or decreased by 2*m* ⋅ π (*m* ∈ Z) while keeping the unit tangent vectors ***T***_*A*_ and ***T***_*B*_ unchanged, the rotation angle *θ* = (*θ*_*B*_ − *θ*_*A*_)/(*k* − 1) can be either positive or negative, and the labeling of the given endpoint-orientation pairs can be swapped. Based on these features, there are different constructions for typical curves under the given conditions. This paper presents a practical method for choosing a suitable curve based on the position relationship between the unit tangent vectors ***T***_*A*_ ***T***_*B*_ and the vector ***P***_*A*_***P***_*B*_.

### Proposed algorithm

Given two endpoint-orientation pairs, the G1 interpolation algorithm is used to find the optimal solution to Eq. (4), where the degree *k* ≥ 2 is an integral and the rotation angle *θ* may have multiple values. *s* > 0 and ‖***V***_0_‖ > 0 are the variables to be determined. The value of *k* can be increased from two to a set maximum value *k*_max_. For a fixed *k*, one must determine the value of *θ* = (*θ*_*B*_ − *θ*_*A*_)/(*k* − 1), which is equivalent to determining Δ*θ* = *θ*_*B*_ − *θ*_*A*_. The proposed algorithm provides a rule for determining Δ*θ* automatically. For case I, let Δ*θ* = *φ*_*A*_ + *φ*_*B*_. For case II, let Δ*θ* = *φ*_*A*_ + *φ*_*B*_ − sign(*φ*_*A*_) ⋅ 2π, where sign(*φ*_*A*_) is the sign of *φ*_*A*_. In this manner, the algorithm can find the typical curve with the lowest degree and smallest total angle between ***T***_*A*_ and ***T***_*B*_.

Once *k* and Δ*θ* are determined, one can use an optimization process to obtain *s* and ‖***V***_0_‖ such that

7 $$ {\displaystyle \begin{array}{l}f\left(s,\left\Vert {\boldsymbol{V}}_0\right\Vert \right)={\left\{{x}_A+\sum \limits_{i=1}^k\left\Vert {\boldsymbol{V}}_0\right\Vert \cdot {s}^{i-1}\cdot \cos \left[{\theta}_A+\left(i-1\right)\cdot \theta \right]-{x}_B\right\}}^2+\\ {}\kern3.75em {\left\{{y}_A+\sum \limits_{i=1}^k\left\Vert {\boldsymbol{V}}_0\right\Vert \cdot {s}^{i-1}\cdot \sin \left[{\theta}_A+\left(i-1\right)\cdot \theta \right]-{y}_B\right\}}^2\end{array}} $$

becomes zero. If *s* > 0, ‖***V***_0_‖ > 0, and Eq. (3) is satisfied, then a typical curve segment can be constructed using these values. Otherwise, the steps of the iteration should be repeated by increasing the degree *k*.

The pseudo code for the proposed algorithm is presented in Algorithm 1.

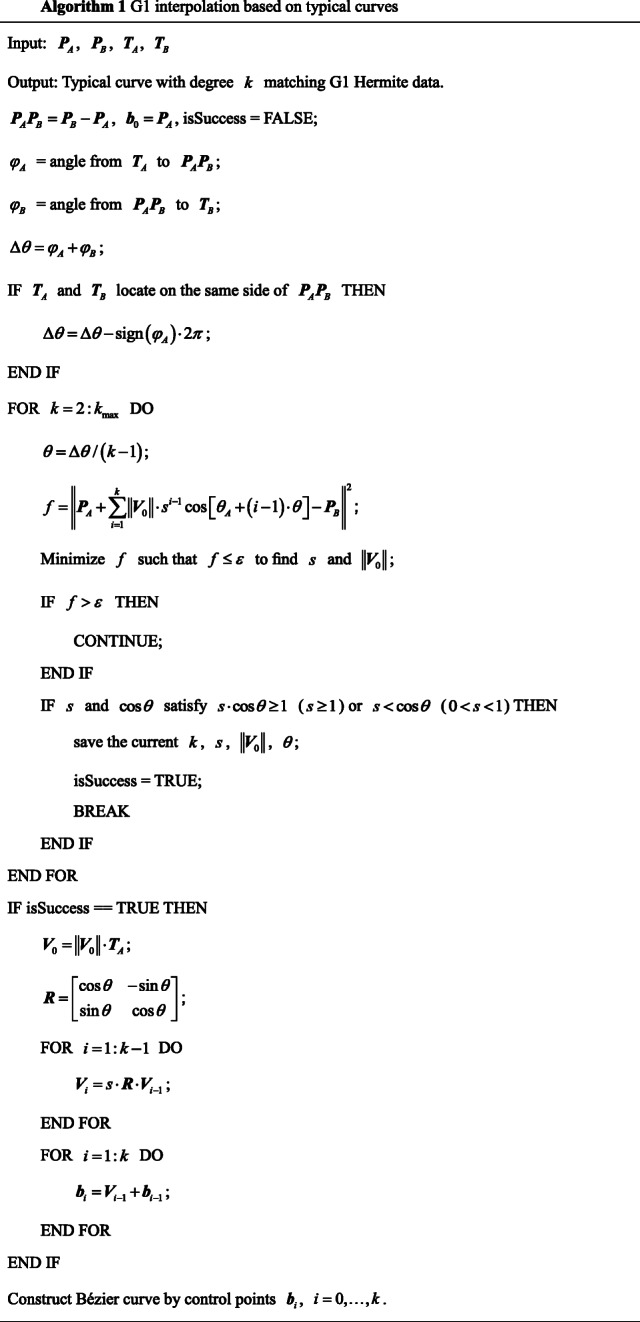



The algorithm first calculates an angle Δ*θ*, which is determined by the position relationship between, ***T***_*A*_, ***T***_*B*_, and ***P***_*A*_***P***_*B*_. Then, Eq. (7) is minimized to a value of zero iteratively by the optimization process. This step was implemented using the interior point method, which can find the minimum value of a constrained nonlinear multivariable function. For the obtained *s* > 0 and ‖***V***_0_‖ > 0, the algorithm checks whether Eq. (3) is satisfied. Once the solutions satisfy Eq. (3), iteration can be stopped and a typical curve can be generated from the obtained data.

The algorithm proposed above avoids outputting multiple solutions by choosing an appropriate rotation angle *θ* = (*θ*_*B*_ − *θ*_*A*_)/(*k* − 1), but this does not mean that there is only one typical curve solution under the given constraints. The main principle of the proposed algorithm is to select the minimum rotation angle Δ*θ* = *θ*_*B*_ − *θ*_*A*_ under the given conditions and then choose the lowest degree *k* to satisfy the constraints. Additional solutions can be obtained by increasing the value of the degree *k*, modifying the angle value Δ*θ* = *θ*_*B*_ − *θ*_*A*_ while leaving the unit tangents ***T***_*A*_ and ***T***_*B*_ unchanged, or even by exchanging the labels of ***P***_*A*_ and ***P***_*B*_ or ***T***_*A*_ and ***T***_*B*_. These methods are discussed in detail in Results and discussion Section.

## Results and discussion

This section presents some numerical experiments based on the proposed algorithm and discusses the existence of multiple solutions.

### Examples of two cases of constraints

This section presents different results for two cases of constraints.

#### Example 1

**Fig. 7:**
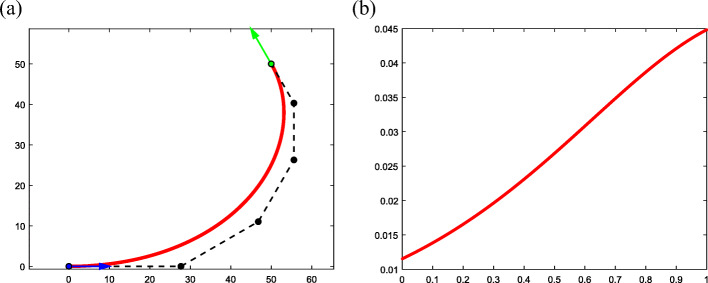
An instance of case I. **a**: Typical curve obtained by the proposed algorithm; **b**: Corresponding curvature plot

Consider data ***P***_*A*_ = (0, 0, 1)^T^, *θ*_*A*_ = 0, ***T***_*A*_ = (1, 0, 0)^T^, ***P***_*B*_ = (50, 50, 1)^T^, ${\theta}_B=\frac{2\uppi}{3}$, and ${\boldsymbol{T}}_B={\left(-\frac{1}{2},\frac{\sqrt{3}}{2},0\right)}^{\mathrm{T}}$ (Fig. 7). It is easy to verify that this is an instance of case I that satisfies the ASC condition. In the proposed algorithm, Δ*θ* = *θ*_*B*_ − *θ*_*A*_ is chosen as $\Delta \theta =\frac{2\uppi}{3}$ and a solution is obtained when the degree *k* = 5 (Fig. 7). Figure 7(a) presents the boundary constraints and resulting curve, where ***P***_*A*_ and ***T***_*A*_ are marked in blue, and ***P***_*B*_ and ***T***_*B*_ are marked in green. Figure 7(b) presents an increasing curvature plot. The data obtained from the typical curve are presented in Table 1.

**Table 1:** The specific data of typical curve for Example 1

*k*	*s*	‖***V***_0_‖	*θ*
5	0.797364	27.680619	π/6

#### Example 2

**Fig. 8:**
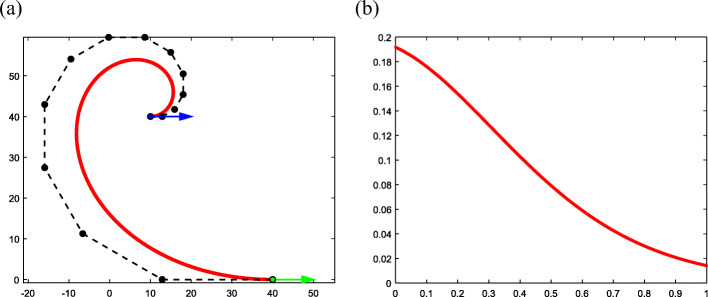
An instance of case II. **a**: Typical curve obtained by the proposed algorithm; **b**: Corresponding curvature plot

Consider data ***P***_*A*_ = (10, 40, 1)^T^, *θ*_*A*_ = 0, ***T***_*A*_ = (1, 0, 0)^T^, ***P***_*B*_ = (40, 0, 1)^T^, *θ*_*B*_ = 0, and ***T***_*B*_ = (1, 0, 0)^T^ (Fig. 8). This is an instance of case II, where Δ*θ* is chosen as Δ*θ* = 2π and one solution is found when *k* = 13 (Fig. 8). The resulting curve satisfies *s* > 1 (Fig. 8(a)), so its curvature plot decreases (Fig. 8(b)). The specific data are presented in Table 2.

**Table 2:** The specific data of typical curve for Example 2

*k*	*s*	‖***V***_0_‖	*θ*
13	1.204772	2.900292	π/6

### Discussion of multiple solutions

Under the given constraints, there are three main reasons for generating multiple solutions: an increase in the degree *k*, different chosen values of Δ*θ*, and the exchange of labels of two endpoint-orientation pairs. Examples of these cases are discussed below.

#### Increasing the degree *k*

**Fig. 9:**
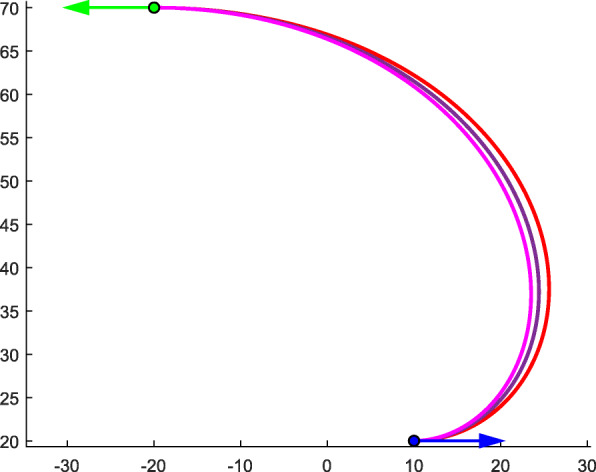
Multiple solutions of typical curves with different degrees under the same conditions

According to Corollary 1, multiple solutions with different degrees exist. Figure 9 presents three typical Bézier curves with different degrees *k* under the given boundary conditions, where ***P***_*A*_ = (10, 20, 1)^T^, *θ*_*A*_ = 0, ***T***_*A*_ = (1, 0, 0)^T^, ***P***_*B*_ = (−20, 70, 1)^T^, *θ*_*B*_ = *π*, and ***T***_*B*_ = (−1, 0, 0)^T^. The three curves in different colors represent three typical curves with different degrees *k*. With an increase in *k*, the arguments of the typical curves trend toward the limits *s* → 1 and *θ* → 0 (Table 3).

**Table 3:** The specific data of typical curves with different degree

Color	*k*	*s*	‖***V***_0_‖	*θ*
Red	6	1.264905	8.780173	π/5
Purple	9	1.189868	4.753024	π/8
Carmine	20	1.090728	1.704246	π/19

#### Different values of Δ*θ*

**Fig. 10:**
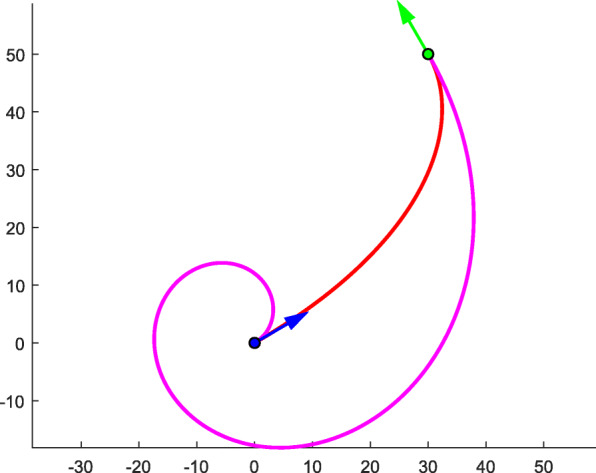
Two typical curves with different Δ*θ* values

The proposed algorithm provides a criterion for the determination Δ*θ*. This is a practical rule. In fact, one can add 2*m* ⋅ π(*m* ∈ Z) to the tangent vector angle while keeping the tangent direction unchanged. Therefore, one can obtain new solutions that satisfy G1 interpolation by adding 2*m* ⋅ π(*m* ∈ Z) to the Δ*θ* selected by the previously proposed algorithm. Figure 10 presents two typical curves with different Δ*θ*, both meeting the given conditions of ***P***_*A*_ = (0, 0, 1)^T^, ${\theta}_A=\frac{\uppi}{6}$, ${\boldsymbol{T}}_A={\left(\frac{\sqrt{3}}{2},\frac{1}{2},0\right)}^{\mathrm{T}}$, ***P***_*B*_ = (30, 50, 1)^T^, ${\theta}_B=\frac{2\uppi}{3}$, and ${\boldsymbol{T}}_B={\left(-\frac{1}{2},\frac{\sqrt{3}}{2},0\right)}^{\mathrm{T}}$. The Δ*θ* value of the carmine curve is 2π greater than that of the red curve (Table 4).

**Table 4:** The specific data of typical curves with different Δ*θ*

Color	Δ*θ*	*k*	*s*	‖***V***_0_‖	*θ*
Red	π/2	3	0.604212	35.719770	π/4
Carmine	5π/2	29	1.081436	1.759416	5π/56

#### Exchange of two endpoint-orientation pairs

**Fig. 11:**
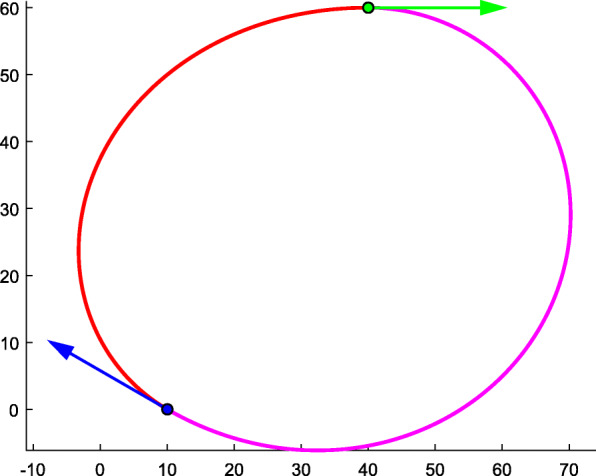
Different typical curves with the exchange of two endpoint-orientation pairs

The construction process of typical curves depends on the sequence of endpoints. If one exchanges the labels of endpoint-orientation pairs, one can obtain different solutions that meet the given conditions. As shown in Fig. 11, the red and carmine curves are two different typical curves that match the same G1 constraint. The difference between the two results is that the red curve takes the blue endpoint-orientation as the starting point and the green endpoint-orientation as the ending point. This means that ***P***_*A*_ = (10, 0, 1)^T^, ${\theta}_A=\frac{5\uppi}{6}$, ${\boldsymbol{T}}_A={\left(-\frac{\sqrt{3}}{2},\frac{1}{2},0\right)}^{\mathrm{T}}$, ***P***_*B*_ = (40, 60, 1)^T^, *θ*_*B*_ = 0, ***T***_*B*_ = (1, 0, 0)^T^, and $\Delta \theta =-\frac{5\uppi}{6}$. The labels of the carmine curve are reversed, meaning ***P***_*A*_ = (40, 60, 1)^T^, *θ*_*A*_ = 0, ***T***_*A*_ = (1, 0, 0)^T^, ***P***_*B*_ = (10, 0, 1)^T^, ${\theta}_B=\frac{5\uppi}{6}$, ${\boldsymbol{T}}_B={\left(-\frac{\sqrt{3}}{2},\frac{1}{2},0\right)}^{\mathrm{T}}$, and $\Delta \theta =-\frac{7\uppi}{6}$. The specific data for these two typical curves are presented in Table 5.

**Table 5:** The specific data of typical curve by exchanging the two endpoint-orientation pairs

Color	Δ*θ*	*k*	*s*	‖***V***_0_‖	*θ*
Red	−5π/6	8	1.091423	8.905500	−5π/42
Carmine	−7π/6	17	1.026836	6.522758	−7π/96

### Curve completion

Curve completion is a geometric continuation of the boundaries of objects that are temporarily interrupted by occlusion [2]. Curve completion requires a visually pleasing shape and is similar to G1 interpolation, but more expansive. The principles for finding an optimal completion curve include minimizing the total curvature square [36] and minimizing the total curvature variation [2]. In this section, these rules are simplified to realize curve completion by achieving monotonic curvature. Two sets of experimental results are presented to demonstrate the application of the proposed method to curve completion and discuss the differences between the proposed approach and other interpolation methods.

**Fig. 12:**
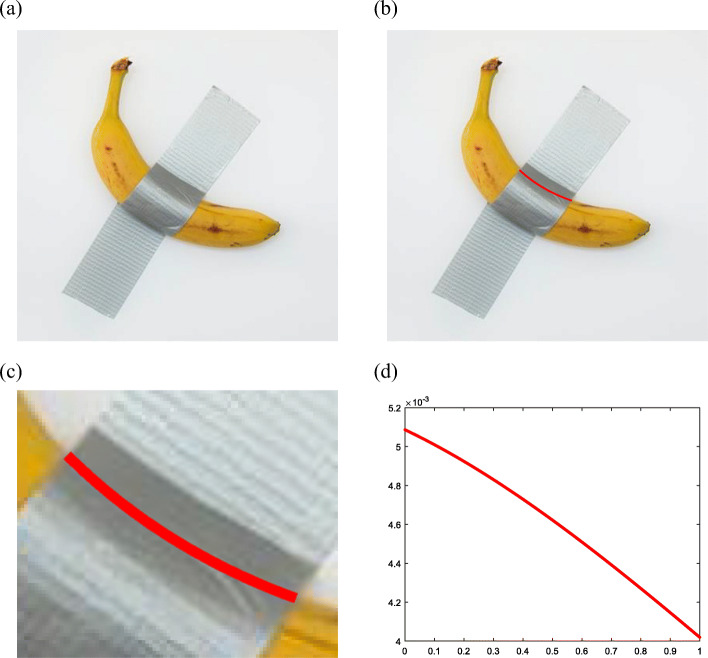
Banana border completion. **a**: Partially occluded banana; **b**: Typical curve; **c**: Local zoomed-in view; **d**: Curvature plot

Figure 12(a) presents a banana that is partially covered by a piece of tape. Figure 12(b) presents the completed curve drawn by the proposed method and Fig. 12(c) presents a local zoomed-in view of the completed curve. Figure 12(d) presents the increasing curvature.

**Fig. 13:**
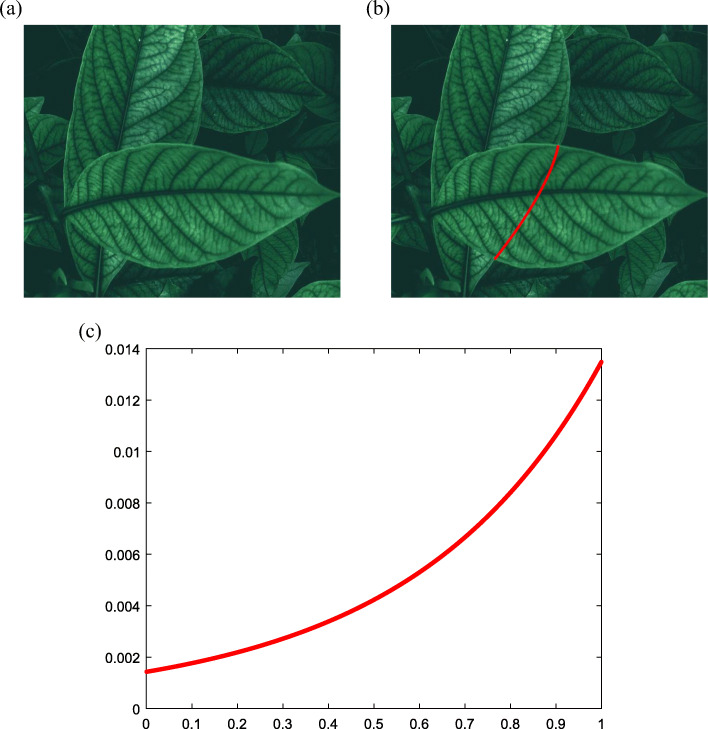
Occluded leaf edge completion. **a**: Two overlapping leaves; **b**: Typical curve; **c**: Curvature plot

Figure 13(a) presents two overlapping leaves, one of which is partially occluded by the other. The proposed method completes the occluded boundary using a typical curve, as shown in Fig. 13(b). The corresponding monotonic curvature is presented in Fig. 13(c).

For the curve completion problem, many studies and methods have been discussed, including methods based on Euler spirals [2, 8, 10], Euler arc splines [3], and cubic B-spline curves [37], but these methods have their own disadvantages. The Euler spiral is expressed by transcendent equations, which may lead to low computational efficiency. Another shortcoming of the Euler spiral is incompatibility with NURBS. A typical curve is a polynomial curve in the Bézier form and the G1 interpolation problem can be transformed into a simple system of nonlinear equations.

An Euler arc spline [3], which consists of several arcs with the same arc length, is considered to be an extension of the Euler spiral. Although it can be represented by NURBS, the continuity at the internal connecting points is only G1 continuous, rather than continuous curvature. In contrast, the curvature of a typical curve is continuous and monotonous over the entire curve.

For the cubic B-spline curve with monotonic curvature mentioned in ref. [37], with an increase in the number of control vectors, the B-spline curve gradually converges to a straight line as the parameter trends toward one. This feature makes the bending curvature inadequate and leads to poor control flexibility. Furthermore, the algorithm for curve completion using a monotonically cubic B-spline curve is a brutal algorithm and the cases of boundary conditions have not been fully elucidated. In the proposed method, the bending curvature can be controlled well based on the rotation angle *θ* or degree *k*, and the construction of a typical curve is simpler than the cubic B-spline curve constructed in ref. [37]. Additionally, the proposed algorithm can handle more constrained cases, which makes it more widely applicable.

## Conclusions

This paper proposed a novel algorithm for G1 interpolation based on typical curves. G1 interpolation was converted into a system of nonlinear equations and a sufficient condition was provided to determine whether there is a typical curve solution with monotonic curvature in advance. The solution is obtained by means of an optimization process and numerical examples demonstrated the effectiveness and practicability of the proposed algorithm.

A single typical curve can only be ‘C’ shaped, but it would be useful to extend the proposed method to ‘S’-shaped curves and apply it to the G2 interpolation problem. In industrial production, applications with 3D curves are valuable. Future work will involve constructing 3D curves with monotonic curvatures.

## Data Availability

Not applicable.

## Abbreviations

CAD: Computer-aided design
